# Membrane Fouling Prediction Based on Tent-SSA-BP

**DOI:** 10.3390/membranes12070691

**Published:** 2022-07-04

**Authors:** Guobi Ling, Zhiwen Wang, Yaoke Shi, Jieying Wang, Yanrong Lu, Long Li

**Affiliations:** 1 College of Electrical and Information Engineering, Lanzhou University of Technology, Lanzhou 730050, China; guobi_ling@163.com (G.L.); yaoke_shi@163.com (Y.S.); jenying0503@163.com (J.W.); yanr_lu@163.com (Y.L.); lilong_zt@163.com (L.L.); 2 Key Laboratory of Gansu Advanced Control for Industrial Processes, Lanzhou University of Technology, Lanzhou 730050, China; 3 National Demonstration Center for Experimental Electrical and Control Engineering Education, Lanzhou University of Technology, Lanzhou 730050, China; 4 GS-Unis Intelligent Transportation System & Control Technology Co., Ltd., Lanzhou 730050, China

**Keywords:** MBR, membrane flux prediction, tent chaotic mapping, SSA, Tent-SSA-BP model

## Abstract

In view of the difficulty in obtaining the membrane bioreactor (MBR) membrane flux in real time, considering the disadvantage of the back propagation (BP) network in predicting MBR membrane flux, such as the local minimum value and poor generalization ability of the model, this article introduces tent chaotic mapping in the standard sparrow search algorithm (SSA), which improves the uniformity of population distribution and the searching ability of the algorithm (used to optimize the key parameters of the BP network). The tent sparrow search algorithm back propagation network (Tent-SSA-BP) membrane fouling prediction model is established to achieve accurate prediction of membrane flux; compared to the BP, genetic algorithm back propagation network (GA-BP), particle swarm optimization back propagation network (PSO-BP), sparrow search algorithm extreme learning machine(SSA-ELM), sparrow search algorithm back propagation network (SSA-BP), and Tent particle swarm optimization back propagation network (Tent–PSO-BP) models, it has unique advantages. Compared with the BP model before improvement, the improved soft sensing model reduces MAPE by 96.76%, RMSE by 99.78% and MAE by 95.61%. The prediction accuracy of the algorithm proposed in this article reaches 97.4%, which is much higher than the 48.52% of BP. It is also higher than other prediction models, and the prediction accuracy has been greatly improved, which has some engineering reference value.

## 1. Introduction

In recent years, with the rapid economic development and industrial progress that has been seen, water resource fouling has deteriorated dramatically, sewage treatment appears to be particularly important, environmental science for sewage treatment requirements and its provisions has also been greatly valued, and the research on sewage treatment is increasing [1,2]. In the process of sewage treatment, improving the efficiency of sewage treatment, realizing green environmental protection, saving energy, reducing consumption and production costs, and improving economic benefits are crucial to the field of sewage treatment [3,4]. As an important means in sewage treatment engineering, membrane bioreactor technology has attracted much attention. The membrane bioreactor (MBR) came into being under the situation of increasingly severe water pollution and increasing demand for treatment efficiency and effluent quality. MBR is a new type of wastewater treatment system combining membrane technology and biological treatment technology, mainly composed of membrane modules and bioreactors. Compared with the traditional sewage treatment process, MBR has the advantages of good and stable outflow water quality, low sludge output, compact equipment and no large amount of space [5,6]. At present, the study of MBR and the prediction of membrane fouling are one of the important topics in the field of sewage treatment. However, the membrane fouling problem seriously affects the permeability and lifetime of the membrane, Membrane fouling will increase the operating cost of membrane bioreactors and become a bottleneck problem limiting the wide application of MBR. Numerous experimental studies have shown that membrane contamination seriously decrease membrane flux, which affects the performance of the MBR process, and results in a larger MBR energy consumption, so the membrane flux value is an important characterization of the degree of membrane contamination [7,8]. Therefore, whether the membrane flux can be predicted in a timely manner and accurately is the key to the control of membrane fouling.

The degree of membrane fouling is related to the value of the membrane flux. In practical applications, the fouling status of the membrane can be analyzed by predicting the value of the membrane flux. However, the membrane flux cannot be directly measured and is affected by the variables of the sewage treatment process. In order to achieve membrane permeation for the detection of the rate, researchers have conducted a lot of research. Chellam [9] and Al-Zoubi et al. [10] obtained multiple process variables related to membrane permeability by analyzing the mechanisms, and 29 of them were selected as auxiliary variables to establish a soft sensing model of membrane permeability based on back propagation neural network (BPNN). The prediction accuracy reached 70%. However, due to the selection of too many auxiliary variables and the large initial scale of the network, the learning time of the network was very long. Too many auxiliary variables also led to the poor anti-interference ability of the network. Therefore, it can only be used in the pilot platform, and cannot be applied in the practical application of wastewater treatment plants. Martínl et al. [11] proposed a general mathematical model to estimate membrane permeability and corrected the model parameters with the actual measurements of the process variables. This model is advantageous due to fewer parameters involved and a simple correction process. It has been widely used in the calculation of membrane permeability. However, the parameters included in this model are rather inaccessible for online corrections, and the prediction accuracy is low. Hwang et al. [12] have successfully predicted the variation trend of membrane flux by studying the correlation between membrane flux and several variables in membrane filtration. However, membrane fouling is a complex and dynamic process, and the numerous factors affecting membrane permeability and their mutual interactions make it difficult to describe membrane permeability with a simple variable relationship.

In recent years, describing membrane contamination process based on intelligent simulation model has become a research hotspot of MBR simulation system. More and more scholars pay attention to the method of predicting membrane flux using intelligent algorithms. For the prediction of membrane fouling, many scholars have proposed different intelligent prediction methods. Through mechanism analysis, Han et al. [13] obtained 26 process variables related to membrane flux. After the selection of six auxiliary variables by the partial least squares (PLS) algorithm, we establish a soft sensor model based on the recursive radial basis function neural network (RRBFNN), and randomly selected 150 sets of data to train the network model, and 80 sets of data were selected, used to verify the accuracy of the prediction model. The prediction accuracy reaches 86.90%. However, there are too few training model arrays, and only 150 sets of data are used for network training, which made it easy to underfit. The model does not fit the training data well, and it does not fit the data well on the test data set, which makes the fitted function not meet the training set, thus affecting the prediction accuracy of the model. Liu et al. [14] study a particle swarm optimization back propagation network (PSO-BP) model: the particle swarm optimization algorithm is used instead of the traditional gradient descent algorithm. The results show that the optimized model has a higher prediction accuracy, and the average error decreased from 2.35% to 0.83%. However, the combination of optimization algorithm model building and the parameter optimization process takes a lot of time, which limits its application scope. Mirbagheri et al. [15] used multi-layer perceptron and radial basis function artificial neural networks (MLPANN and RBFANN) to predict trans-membrane pressure (TMP) and membrane flux, and used the genetic algorithm (GA) algorithm to optimize the weights. The results show that the optimized model has a higher prediction accuracy. However, because the dynamic process of sewage treatment is complex, the change of membrane flux cannot be expressed by a simple static mapping relationship.

At present, swarm intelligence optimization algorithms such as the sparrow search algorithm (SSA) have been widely used in engineering problems. The sparrow search algorithm (SSA) is a new metaheuristic algorithm proposed by Xue and Shen in 2020 [16]. Liu et al. [17] established a prediction model based on sparrow-search-algorithm-optimized support vector machine regression (SSA-SVR) to predict the settlement of coal gangue roadbed of a Shao Expressway in Hunan Province and compared the prediction results with particle swarm optimization-support vector regression (PSO-SVR) and genetic algorithm-support vector regression (GA-SVR) models. The results show that the SSA-SVR prediction model has high accuracy and good generalization ability. Liu et al. [18] proposed a combined prediction model based on the Sparrow Algorithm (SSA) to optimize the extreme learning machine, aiming at the instability of the extreme learning machine (ELM) model and the inaccuracy of the forecast results. The weights and thresholds of ELM are optimized by using SSA with a fast convergence speed, high precision and good stability, and the accurate prediction of wind power is realized.

However, SSA, like other population intelligence optimization algorithms, still suffers from the problems of decreasing population diversity and easily falling into local optimality when its search is close to the global optimum. Considering the ergodic uniformity and fast convergence of tent map, this study proposes an improved sparrow search algorithm (Tent-SSA). In order to effectively select auxiliary variables, establish a suitable membrane flux prediction model, and realize the online accurate prediction of membrane flux, firstly, the mechanism of membrane fouling factors is analyzed in this paper, and secondly, principal component analysis (PCA) is applied. Select auxiliary variables, and then introduce tent mapping to initialize the population to increase population diversity. Finally, the Tent-SSA-BP neural network was combined to establish a Tent-SSA-BP membrane flux soft-sensing model, and the simulation data of Seong-Hoon Yoon’s spreadsheet model was used to verify the reliability and stability of the model and to prediction of membrane flux value [19].

## 2. Theory Related to BP Neural Networks

**Figure 1:**
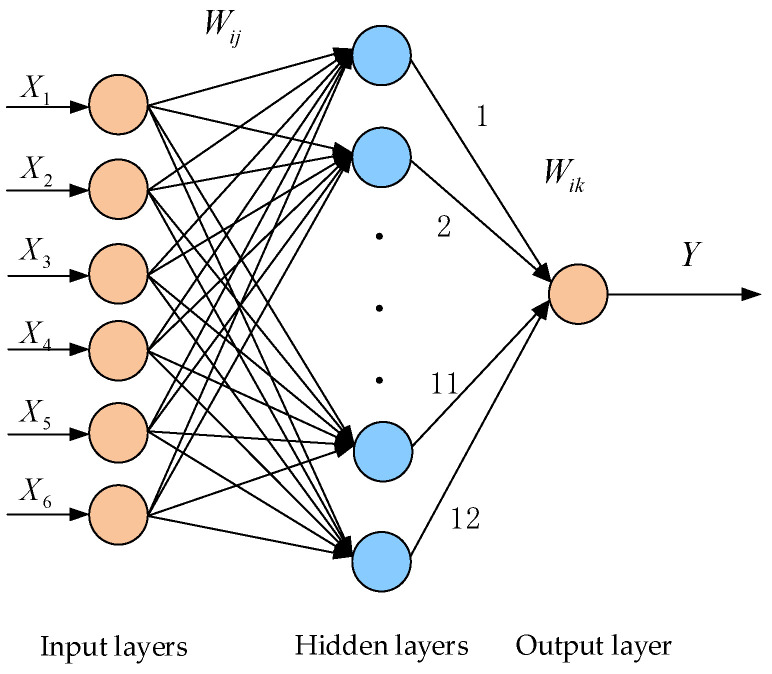
BP network 6-12-1 structure diagram.

The BP neural network has the ability of self-organization, self-learning and self-adaptation, and its principle is simple and easy to implement [20]. It has been widely used in many fields. The network structure diagram of BP network 6-12-1 is shown in Figure 1.

(1) $$\omega_{ij}(i+1)=\omega_{ij}(i)-\mu \frac{\partial E}{\partial \omega_{ij}}\;(\mu >0)$$

The learning process of the BP algorithm is based on the gradient descent method to correct network weights and thresholds, which minimizes the sum of squared network errors, where *E* is the total network error. The error reduction is carried out in the direction of the negative gradient, and it is easy to get into the dilemma of local minimum value. In order to make the deviation between the actual output and the target value of each unit smaller than the specified value, the connection weights need to be constantly adjusted. When there are too many training samples or the relationship between input and output is complex, the convergence speed of the network will become slow [21]. In addition, the BP algorithm has other limitations, so this article uses the improved SSA algorithm to optimize the BP network.

## 3. Sparrow Search Algorithm

### 3.1. Standard SSA

The idea of SSA is derived from the foraging and anti-predatory behaviors of sparrow populations and can be abstracted as an explorer–follower–warner model [22]. The Explorer has a high energy reserve and a high fitness value, which mainly provides foraging areas and directions for followers. Followers follow the explorer with the best fitness value to find food to gain their own energy reserves and increase their fitness value [23,24]. Some followers may also constantly monitor the explorer, compete for food, and alert when they are aware of the danger and move quickly to safe areas to get a better location, while sparrows in the middle of the population walk randomly close to other sparrows, known as anti-predatory behavior [25,26]. At the same time, if the alert value is greater than the security threshold, the explorer needs to take all followers out of the danger zone.

In SSA, if there are N sparrows in a D-dimensional search space, the location of each sparrow is shown in Formula (2):

(2) $$\begin{array}{l} X=\left(\begin{array}{cccccc} x_{1,1} & x_{1,2} & \ldots & x_{1,d} & \ldots & x_{1,D}\\ x_{2,1} & x_{2,2} & \vdots & x_{2,d} & \vdots & x_{2,D}\\ \vdots & \vdots & \vdots & \vdots & \vdots & \vdots \\ x_{i,1} & x_{i,2} & \ldots & x_{i,d} & \ldots & x_{i,D}\\ \vdots & \vdots & \vdots & \vdots & \vdots & \vdots \\ x_{N,1} & x_{N,2} & \ldots & x_{N,d} & \ldots & x_{N,D} \end{array}\right) \end{array}$$

In the formula, i=1,2,…,N, d=1,2,…,D, and $x_{id}$ indicates the position of the i-th sparrow in the d-th dimension.

Since the explorer guides the movement of the whole sparrow population, and can find food anywhere, its location is updated as follows:

(3) $$x_{i,d}^{t+1}=\left\{ \begin{array}{ll} x_{i,d}^{t}\cdot \exp\left(\frac{-i}{a\cdot iter_{\max}}\right), & R_{2}<ST\\ x_{i,d}^{t}+Q\times L, & R_{2}\geq ST \end{array}\right.$$

In the formula, t represents the current number of iterations, $iter_{\max}$ is the maximum number of iterations, a is a random number with an interval (0, 1], Q is a random number with a normal distribution, and L represents a matrix of 1×d, where each element is 1 and $R_{2}\in \left[0,1\right]$ represents the alert value; ST∈[0.5,1] represents a security threshold. When $R_{2}<ST$, it means there are no predators around, and the explorer will enter a wider search mode; if $R_{2}\geq ST$, it means that some sparrows have found predators and all need to fly quickly to other security zones.

Followers follow the explorer in search of food and may compete with the explorer for food to increase their own predation rate. We can update formula to:

(4) $$x_{i,d}^{t+1}=\left\{ \begin{array}{ll} Q\cdot \exp\left(\frac{xworst_{d}^{t}-x_{i,d}^{t}}{a\cdot iter_{\max}}\right), & i>N/2\\ xbest_{d}^{t+1}+\frac{1}{D}\sum_{d=1}^{D}\left(\left|x_{i,d}^{t}-xbest_{d}^{t+1}\right|rand\left\{-1,1\right\}\right), & i\leq N/2 \end{array}\right.$$

In the formula, $xworst_{d}^{t}$ represents the lowest overall position of sparrows in the d-th dimension during the t-th iteration, and $xbest_{d}^{t}$ represents the lowest overall position of sparrows in the d-th dimension during the t+1-th iteration. When i>N/2, the i-th follower with poor fitness was most likely to starve; otherwise, the i-th follower will randomly find a place near the best location for the explorer to feed.

Assuming that the early warning sparrow accounts for about 10~20% of the sparrow population, its initial position is randomly determined, and the mathematical model can be expressed as:

(5) $$x_{i,d}^{t+1}=\left\{ \begin{array}{ll} xbest_{d}^{t}+\beta \cdot \left|x_{i,d}^{t}-xbest_{d}^{t}\right|, & f_{i}\neq f_{g}\\ x_{i,d}^{t}+k\cdot \left(\frac{\left|x_{i,d}^{t}-xworst_{d}^{t}\right|}{\left|f_{i}-f_{w}\right|+\varepsilon }\right), & f_{i}=f_{g} \end{array}\right.$$

In the formula, β is a random normal distribution with a mean of 0 and a variance of 1, which is used as a step control parameter, k is a random number between [−1, 1], $f_{i},\;f_{g},\;f_{w}$ represents the fitness value of the current sparrow, the current global best fitness value, and the worst fitness value, respectively, and ε is the minimum constant to avoid zero-point error. $f_{i}\neq f_{g}$ means sparrows are at the edge of the population, when $f_{i}=f_{g}$, sparrows in the middle of the population are aware of the danger and need to move elsewhere.

### 3.2. Improved SSA

#### 3.2.1. Tent Chaotic Mapping

Chaotic phenomena refer to the existence of random and irregular variables in a certain system that are not repeatable, predictable and indeterminate [27]. At present, the common chaotic operators are logistic mapping function operator and tent chaotic mapping function operator. Logistic mapping function is a chaotic system, but the speed of searching for the best solution is limited by the asymmetry of its function distribution [28,29,30].

**Figure 2:**
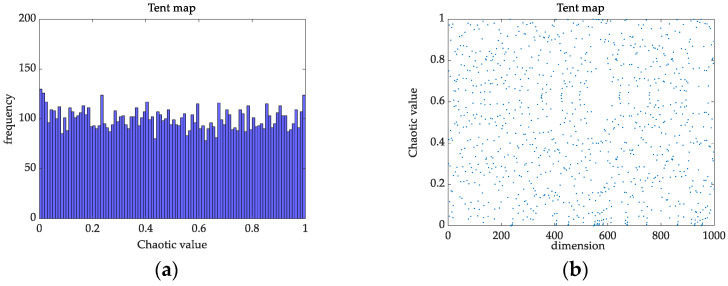
Tent chaotic sequence. (**a**) Histogram; (**b**) Distribution map.

**Figure 3:**
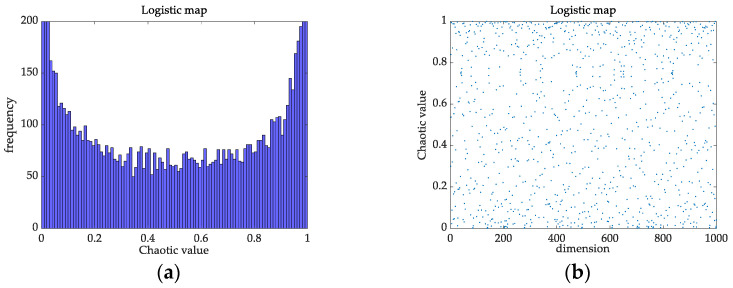
Logistic chaotic sequence. (**a**) Histogram; (**b**) Distribution map.

At present, many scholars have introduced chaos into SSA [31]. The heterogeneity of logistic chaotic mapping has a greater impact on the search speed and accuracy, while tent chaotic mapping has better traversal uniformity and faster search speed than logistic chaotic mapping. From the histograms and distributions of the tent chaotic sequence in Figure 2, and logistic chaotic sequence in Figure 3, we can see that the frequency of the mapping of the logistic chaotic sequence in the range [0, 0.05] and [0.95, 1] is higher than that of the other segments, while the distribution of the tent chaotic sequence mapping in the [0, 1] interval is more uniform and the iteration speed is faster. When logistic chaotic mapping is applied to the initialization of a population, the heterogeneity of its chaotic sequence results in an uneven distribution of the initialization population, which affects the speed and accuracy of the algorithm in the optimization process. This article uses tent’s traversal to generate a uniformly distributed chaotic sequence, which reduces the influence of initial values on algorithm optimization.

The tent chaotic mapping function is also known as the tent mapping function, which is calculated as Formula (6).

(6) $$x_{i+1}=\left\{ \begin{array}{ll} 2x & 0\leq x_{i}<1/2\\ 2\left(1-x_{i}\right) & 1/2\leq x_{i}\leq 1\; \end{array}\right.$$

The tent mapping function requires a bernoulli shift transformation. In the [0, 1] interval, the computer performs a tent mapping to left-shift the binary number of the fractional part. This transformation is suitable for computing large-scale data. The bernoulli shift transformation is shown in Formula (7).

(7) $$x_{i+1}=\left(2x_{i}\right)\mathrm{mod}1$$

The chaotic sequence generated by tent chaotic mapping in the [0, 1] interval is periodic, but there are also unstable periodic points. Because tent chaotic sequence iteration may fall into small and unstable periodic points, a random variable rand(0,1)×1/N needs to be added to the initial tent chaotic mapping function, which is shown in Formula (8).

(8) $$x_{i+1}=\left\{ \begin{array}{ll} 2x_{i}+\mathrm{rand}\left(0,1\right)\times \frac{1}{N}, & 0\leq x_{i}<1/2\\ 2\left(1-x_{i}\right)+\mathrm{rand}\left(0,1\right)\times \frac{1}{N}, & 1/2\leq x_{i}\leq 1\; \end{array}\right.$$

The expression after bernoulli shift transformation is shown in Formula (9): N is the number of particles in the tent chaotic sequence.

(9) $$x_{i+1}=\left(2x_{i}\right)\mathrm{mod}1+\mathrm{rand}\left(0,1\right)\times \frac{1}{N}$$

In conjunction with the above description of the tent mapping properties, the basic steps for generating a tent mapping chaotic sequence within a feasible domain are as follows:

Step 1: Randomly generates the initial value $x_{0}$ (be careful not to let $x_{0}$ fall into small cycles), which is marked as group Z, $z_{1}=x_{0}$, i=j=1.

Step 2: Iterate by Formula (9), increasing by 1 each time, resulting in sequence X.

Step 3: If it reaches the maximum number of iterations, it goes to Step 5. Conversely, if it falls into an unstable periodic point, it goes to Step 2. 

Step 4: Change the initial iteration value by formula $x_{i}=z_{j+1}=z_{j}+\varepsilon$; in the formula, ε is a random number, j=j+1,then turns to Step 2. 

Step 5: In the end, saving sequence X generated by the iteration.

#### 3.2.2. Tent Chaotic Mapping SSA

The initial location information of the sparrow population is randomly generated by the standard SSA at the beginning of execution, which results in poor diversity within the sparrow population. As a result, the algorithm finds a solution that is not globally optimal and affects the convergence speed and accuracy of the SSA [32]. We use the tent chaotic mapping function to optimize SSA, and the tent sparrow search algorithm (Tent-SSA) is improved by introducing tent chaotic mapping to initialize SSA. It can improve the search performance of the algorithm and avoid the algorithm falling into local optimum. The steps are as follows:

Step 1: Initialize SSA parameters;

Step 2: Use the tent chaotic mapping function to generate a uniform chaotic sequence, the initial position of members of the sparrow population;

Step 3: Calculate the fitness value of each sparrow to determine the position of the individual with the best and worst fitness value;

Step 4: Determine the number of explorers in the sparrow population and calculate their updated locations based on Formula (3);

Step 5: Determine the number of followers in the sparrow population and calculate their updated positions based on Formula (4);

Step 6: Determine the number of warners in the sparrow population and calculate their updated location based on Formula (5);

Step 7: Calculate each sparrow’s fitness value and update it if the new fitness value is better than the previous one;

Step 8: If the maximum number of iterations of the algorithm is reached, the location information of the sparrow with the best global fitness value is output. Otherwise, go to Step 4 to continue execution.

## 4. Membrane Fouling Prediction Model

### 4.1. Selection of Variables

The properties of membrane components, operation conditions and characteristics of sludge mixtures are the main influencing factors of membrane fouling [33]. The properties of the membrane mainly include membrane materials, surface roughness, and the pore value of the membrane. Operating conditions include temperature, aeration, sludge retention time (SRT), and hydraulic retention time (HRT) [34]. The characteristics of sludge mixtures include total suspended solid (TSS), sludge load, mixed liquor suspended solids (MLSS), microbial products such as soluble microbial products (SMP), and extracellular polymeric substances (EPS). In addition, variables such as flow rate, air–water ratio, water production pressure, viscosity, biochemical oxygen demand (BOD), chemical oxygen demand (COD), hydrogen ion concentration (pH), and oxidation-reduction potential (ORP), which occur during the wastewater treatment process, and dissolved oxygen, nitrate, total phosphorus (TP), effluent turbidity, and dry solid (DS) in the aerobic zone all affect the membrane flux value [35,36]. There are many factors that affect the membrane flux and the relationship is complex, so it is necessary to select appropriate auxiliary variables.

### 4.2. Principal Component Analysis

The principal component analysis (PCA) algorithm is a widely used data dimensionality reduction algorithm. The PCA algorithm can more scientifically and effectively screen out the auxiliary variables with the greatest correlation with membrane flux [37].

Set the research of a certain thing to involve P indicators, which is represented by $x_{1},x_{2},\ldots ,x_{p}$, respectively; these P indicators constitute a random vector of P -dimension as $X=(x_{1},x_{2},\ldots ,x_{p})$. Transform X linearly to form a new synthetic variable, which is represented by Y, that is, the new synthetic variable can be represented linearly by the original variable [38]:

(10) $$\left\{ \begin{array}{l} Y_{1}=u_{11}X_{1}+u_{12}X_{2}+\ldots +u_{1p}X_{p}\\ Y_{2}=u_{21}X_{1}+u_{22}X_{2}+\ldots +u_{2p}X_{p}\\ \ldots \ldots \\ Y_{p}=u_{p1}X_{1}+u_{p2}X_{2}+\ldots +u_{pp}X_{p} \end{array}\right.$$

In the formula, if $u_{i}{}^{\prime }u_{i}=1$, $Y_{i}$ and $Y_{j}$ are independent of each other. The variance of $Y_{1},Y_{2},\ldots ,Y_{p}$ decreases in turn, then $Y_{1},Y_{2},\ldots ,Y_{p}$ are the first, second, …, P -th principal components of the original variable. The specific calculation steps of principal component analysis are as follows:

Step 1: Select the initial analysis variable;

Step 2: Find the principal component from the correlation matrix;

Step 3: Find the eigenvalue of the correlation matrix and the corresponding standard eigenvector;

Step 4: Calculate the variance contribution rate and the cumulative variance contribution rate of each principal component and select the principal component.

In this article, we use the membrane flux as a predictor; combining with the specific conditions of the experiment, we pretreat the principal component analysis of the original data when using MATLAB. When reducing the dimension, it is stipulated that 85% of the data information is guaranteed, so that the data loses less data information when reducing the dimension. The values of each index were counted, and normalized and standardized. The index data were analyzed by SPSS, and the eigenvalues and cumulative contribution rates of each principal component were obtained as shown in Table 1. 

The eigenvalues of the first three principal components are all greater than 1, and the cumulative variance contribution rate is 89.308%, which has a large contribution to the explanatory variables. Most of the information affecting membrane fouling is included, so the first three are extracted as principal components. Taking the ratio of the variance contribution rate corresponding to each principal component to the cumulative contribution rate as the weight, the calculation of the sustainability index is shown in Formula (11).

(11) $$S=0.54Y_{1}+0.21Y_{2}+0.14Y_{3}$$

In the formula, $Y_{1}$, $Y_{2}$ and $Y_{3}$ are the main component factors. Through the analysis of the coefficient matrix of the components, it has been concluded that the main relevant components of $Y_{1}$ are TSS, MLSS and SRT, the main relevant components of $Y_{2}$ are total resistance and TMP, and the main relevant component of $Y_{3}$ is water production pressure.

Finally, we summarize six of the most obvious factors affecting the membrane fouling: TSS, MLSS, total resistance, TMP, SRT and water production pressure.

### 4.3. Establishment of Tent-SSA-BP Prediction Model

**Figure 4:**
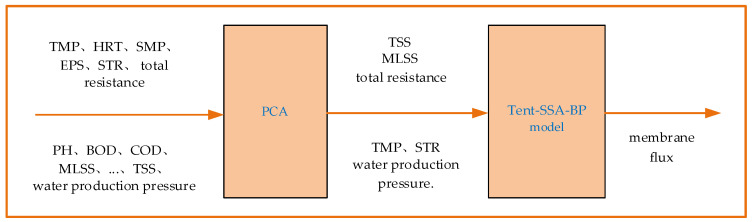
Membrane fouling prediction model.

Membrane flux is an important index parameter reflecting the degree of membrane fouling; therefore, we use it as the output of the model, and we use six factors after the principal component dimension reduction as input. Finally, we establish a prediction model for membrane fouling based on improved SSA (Tent-SSA) and the optimized BP network as shown in Figure 4.

**Figure 5:**
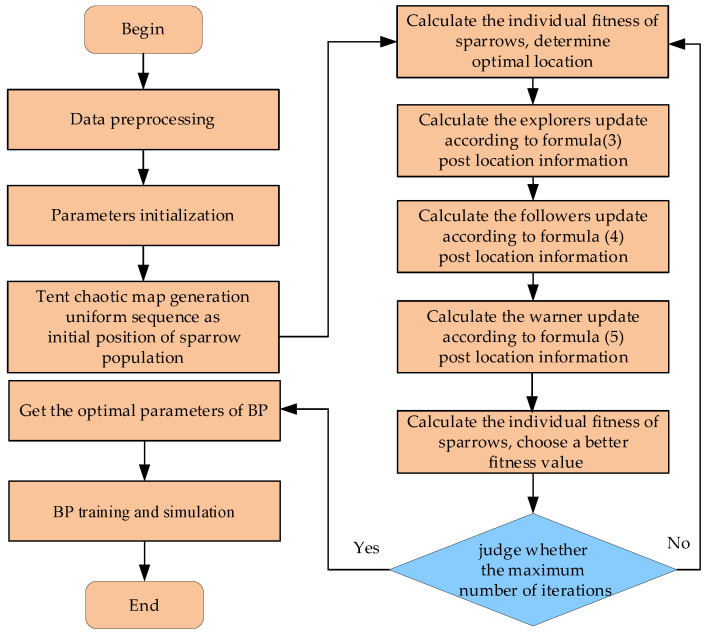
Flowchart of Tent-SSA-BP algorithm.

This article optimizes the learning parameters of the BP network based on SSA. First, we optimize the weights and thresholds of the BP network by using improved SSA; second, we assign these optimization values to the network to get the optimized BP network, tent-SSA-BP network; then, the BP algorithm is used to complete the final network training; finally, we evaluate the performance of the optimized network by simulation data. The algorithm flow is shown in Figure 5, and the algorithm steps are as follows:

Step 1: Auxiliary variable selection and preprocessing, and use PCA for dimension reduction;

Step 2: Initialize relevant parameters, initialize population, introduce tent mapping, and improve initial population distribution uniformity;

Step 3: Compute and sort the fitness values according to the objective function;

Step 4: Use Formulas (4) and (5) to update follower and guardian positions;

Step 5: Determine whether to stop, execute exit or continue cycle;

Step 6: Train the BP network and use it for prediction.

### 4.4. Evaluating Indicator

In order to make the output accuracy more intuitive, we introduce the following evaluation indicators.

(1) Mean absolute percentage (*MAPE*)

(12) $$MAPE\;=\frac{1}{n}\sum_{i=1}^{n}\left|\frac{y_{i}-y_{i}^{*}}{y_{i}^{*}}\right|\times 100\%$$

(2) Root mean square error (*RMSE*)

(13) $$RMSE=\sqrt{\frac{\sum_{i=1}^{n}{\left(y_{i}-y_{i}^{*}\right)}^{2}}{n}}$$

(3) Mean absolute error (*MAE*)

(14) $$MAE=\frac{\sum_{i=1}^{n}\left|y_{i}-y_{i}^{*}\right|}{n}$$

In the formula, $y_{i}$ is the true value, $y_{i}^{*}$ is the model output value, n is the number of test samples.

## 5. Experiment and Simulation

### 5.1. Improved Algorithm Performance Test

#### 5.1.1. Benchmark Function

In order to test the optimization performance of the improved Tent-SSA, we select 8 benchmark test functions. As Table 2 shows, the algorithm sets the population value to 50, the proportion of explorers and early warner is 20%, the warning value is 0.6, the maximum iteration number is 100, the dimension is 30, and each runs 20 times independently. Mean and standard deviation are introduced as performance measures in the experiment. Mean reflects the accuracy of the optimization algorithm, while standard deviation reflects the stability and robustness of the algorithm.

#### 5.1.2. Comparison of Different Intelligent Optimization Algorithms

The parameters of computer device used in the experiment are i5-7500HCPU, 2.30 GHz, 16 G running memory, and Windows 10 system. The simulation experiment is carried out on Matlab2018b. The Tent-SSA algorithm proposed in this article is compared with SSA, PSO and whale optimization algorithm (WOA) on eight benchmark functions to obtain the mean and standard deviation of the function. The experimental data (the bolded data are the optimal value for this set) are shown in Table 3.

**Figure 6:**
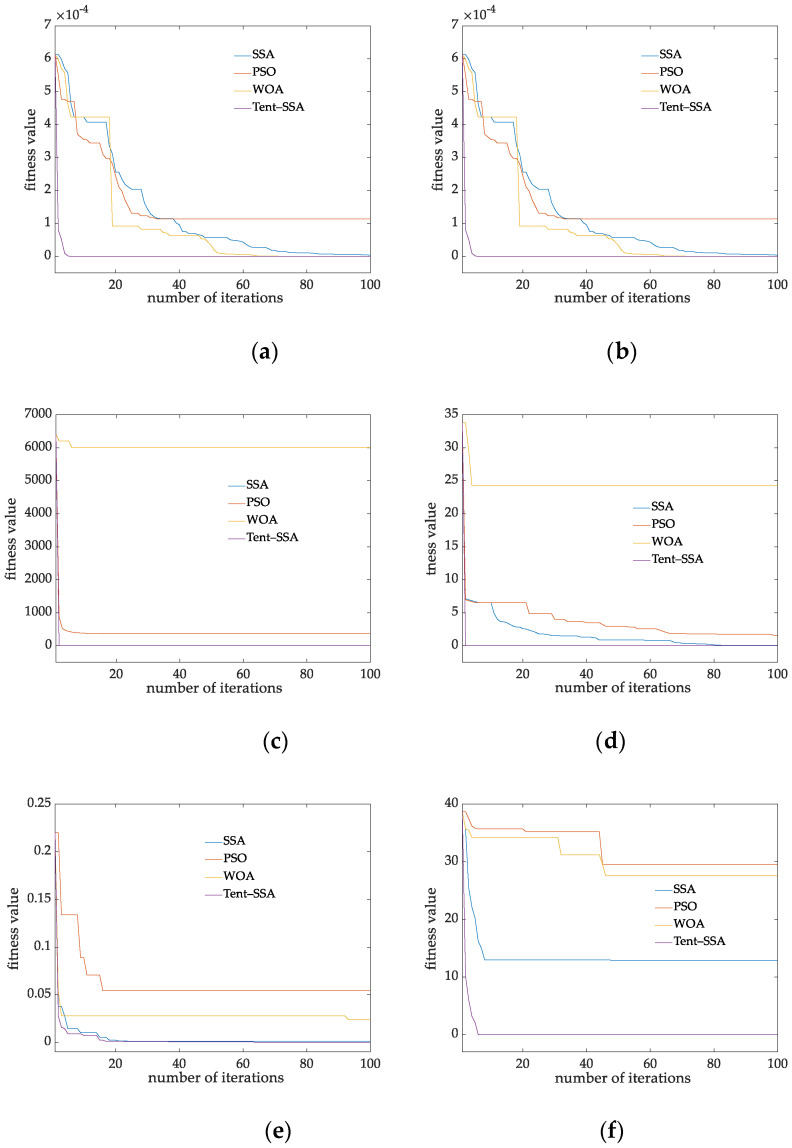

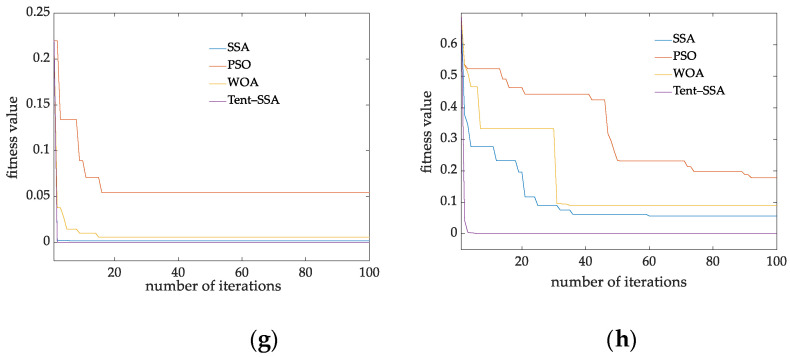
Iterative convergence curves for different test functions. (**a**–**h**) $F_{1}$–$F_{8}$ iterative convergence curves.

It can be seen from Table 3 that for high-dimensional unimodal Function $F_{1}$ to $F_{5}$, the optimization effect of the proposed tent SSA algorithm is significantly better than that of SSA, PSO and WOA, in which the optimization effect for $F_{4}$ reaches 100%, and the optimization effect for $F_{1}$ to $F_{3}$ is dozens of orders of magnitude higher than that of other algorithms and standard deviation. Deviation is generally smaller than that of other algorithms, indicating that the optimization accuracy of tent SSA has been significantly improved and the quality of finding global suboptimal solutions has been improved, and the random error of the algorithm is reduced. It shows that tent SSA improves the population diversity through tent optimization strategy, which makes the algorithm jump out of the local optimal value. In order to more intuitively compare the convergence accuracy and convergence speed of the algorithm, this paper draws the convergence curve of the test function according to the iteration times and fitness values, and obtains the iterative convergence curve of each function, as shown in Figure 6.

Ascan be seen from Figure 6, for the eight benchmark functions, the convergence speed of Tent-SSA in the early stage is greatly improved compared with other intelligent algorithms. It can quickly search the search space and improve its global search ability, thus greatly shortening the exploration cycle of the early algorithm and improving its optimization search performance. At the same time, the algorithm takes into account the optimization accuracy and can be closer to the ideal optimal solution. For function $F_{3}$ and $F_{7}$, the improvement of convergence accuracy of Tent-SSA is not significant compared with SSA. However, compared with SSA, Tent-SSA improves the convergence speed while taking into account the optimization accuracy of the algorithm.

### 5.2. Experimental Results and Analysis

The experimental data in this article come from a spreadsheet model of Seong-Hoon Yoon. The data related to membrane fouling can be simulated and processed according to the experimental requirements to ensure that the data are closer to the actual data. The subjects of study are all polyvinylidene fluoride (PVDF) hollow microfiltration (MF) components. The water inlet mode is external pressure, and the effective usage area of the membrane is 20 m^2^. In MBR reaction pool, the microorganisms and pollutants in the pool are complex, so the selection of membrane contamination influencing factor largely determines the accuracy of membrane contamination prediction. In order to improve the prediction accuracy, finally, we determine 592 sets of data for model simulation by PCA dimension reduction and normalization: 500 sets of data are used for model training, and the remaining 92 sets are used for model testing.

(1) Comparison of results before and after improving optimization algorithm.

**Figure 7:**
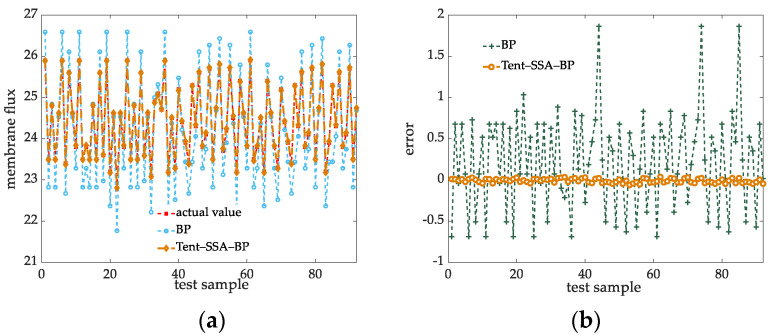
Optimizing algorithm to improve the comparison chart of prediction results. (**a**) Prediction contrast map. (**b**) Error comparison diagram.

The simulation results of the actual operation data are shown in Figure 7.

From the simulation curve in Figure 7a, it can be seen that the deviation between the unmodified BP network and the true value fluctuates greatly, and the improved prediction model has a smaller fluctuation than the true value. From the comparison of prediction errors in Figure 7b, it can be seen that the Tent-SSA-BP model proposed in this article has a greater improvement in prediction errors than the standard algorithm, and the fluctuation range of errors is significantly reduced, which can better reflect the change of membrane flux.

(2) Prediction accuracy of different soft measurement models

**Figure 8:**
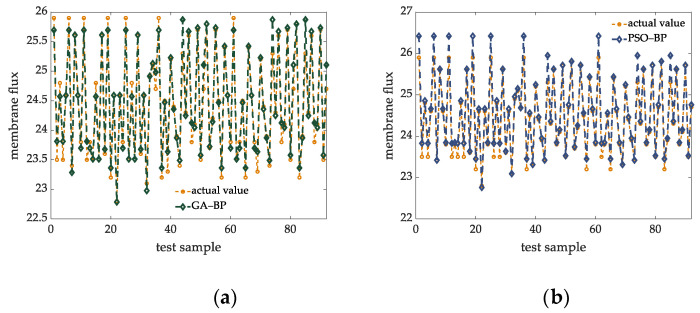

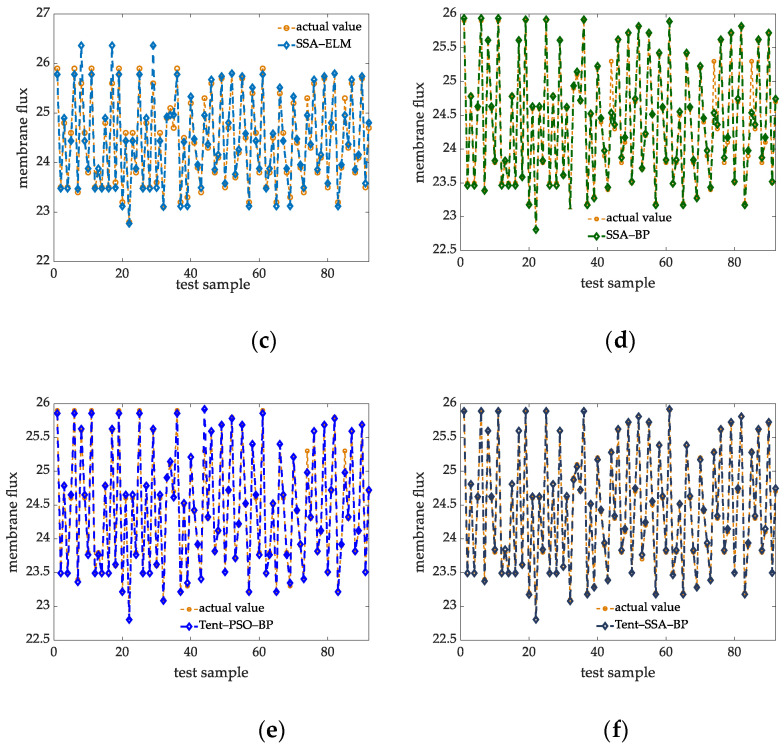
Prediction results of different soft measurement models. (**a**) GA-BP prediction contrast map. (**b**) PSO-BP prediction contrast map. (**c**) SSA-ELM prediction contrast map. (**d**) SSA-BP prediction contrast map. (**e**) Tent-PSO-BP prediction contrast map. (**f**) Tent-SSA-BP prediction contrast map.

In order to compare the prediction accuracy of the soft sensing model proposed in this article, the measurement models are built under the same experimental conditions by using GA-BP, PSO-BP, SSA-ELM, SSA-BP and Tent-PSO-BP models. The prediction results of different soft sensing models are shown in Figure 8. Through the comparison of prediction results, we can find that the prediction results of other methods fluctuate greatly at the peak and trough of the original data, which cannot well reflect the changes of the original data. In this case, the Tent-SSA-BP model proposed in this article is superior to other models to a certain extent. The comparison of prediction errors of each model is shown in Table 4.

**Figure 9:**
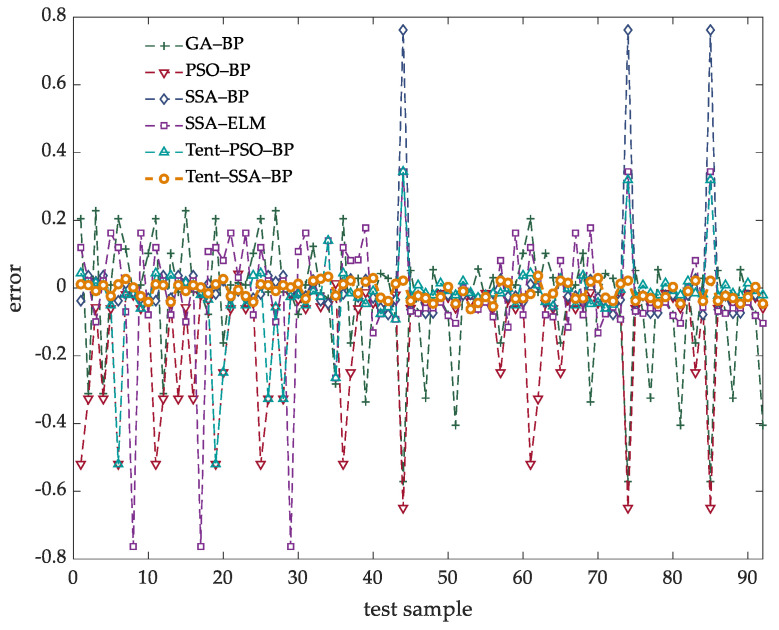
Prediction error curve.

According to the data in Table 4, under the same experimental conditions, compared with the BP model before improvement, the improved soft sensing model reduces MAPE by 96.76%, RMSE by 99.78% and MAE by 95.61%. For SSA-ELM model, MAPE decreased by 84.78%, RMSE decreased by 97.16% and Mae decreased by 80.47%. For SSA-BP model, MAPE is reduced by 70.83%, RMSE is reduced by 95.59% and MAE is reduced by 60.63%. The prediction accuracy of the algorithm proposed in this article reaches 97.4%, which is much higher than 48.52% of BP. It is also higher than of other prediction models, and the prediction accuracy has been greatly improved. According to various indicators, the prediction effect of Tent-SSA-BP soft sensing model is better than that of the unmodified soft sensing model. Compared with other models, the prediction accuracy of the algorithm proposed in this article is the highest, and the prediction error curve is shown in Figure 9.

(3) Variable noise membrane flux prediction results and analysis with different prediction methods

In the actual operation process of MBR, environmental noise exists when the membrane module is treating sewage. At the same time, due to the characteristics of the membrane module, there is also noise, which produces unnecessary randomness in the collection of membrane fouling data. It is very important to add variable noise experiment to the membrane flux prediction, for the simulated data needs to be more in line with the uncertainty of the operation of membrane module under actual working conditions. In this article, aiming at the membrane fouling data as the training sample, we add Gaussian white noise with a signal-to-noise ratio of 4, 8 and 12 dB to the test sample, and compare the obtained membrane fouling prediction results with other prediction methods. The experimental results are shown in Table 5.

It can be seen from the comparative data in Table 5 that the accuracy of several models has decreased in the experimental results of different signal-to-noise ratios, but through longitudinal comparison, the prediction accuracy of membrane flux based on Tent-SSA-BP is higher than that of other methods. In the signal-to-noise ratio interference experiment, it performs better in 4, 8 and 12 dB, and the prediction accuracy remains stable at more than 91%.

## 6. Conclusions

The accurate prediction of membrane fouling has an important impact on the sewage treatment process. To achieve accurate measurement on-line, we propose a Tent-SSA-BP prediction model. First, we optimize the improved SSA, then use the PCA algorithm to reduce the dimension of the auxiliary variables after pretreatment, and we use the improved SSA to optimize the key parameters of the BP network. Finally, we use the optimal parameters to predict the membrane flux. The results are shown as follows:

(1) We use PCA to reduce the initial auxiliary variables. At the same time, we improve the efficiency of the algorithm and reduce the probability of over-fitting. In order to solve the problem that the diversity of population is reduced and the local optimal solution is easily trapped in the later stage of the optimization algorithm, we introduce the tent chaotic map to improve the uniformity of initial population distribution and the ability of the algorithm to find the global optimal solution. Tent-SSA-BP can find the global optimal solution more easily and quickly.
(2) Based on the Tent-SSA-BP method proposed in this article, the prediction model of membrane flux in membrane fouling, whether it is the training speed or prediction accuracy of the model, shows better performance than other methods (GA-BP, PSO-BP, SSA-ELM, SSA-BP, Tent-PSO-BP), which is more suitable for the prediction model of membrane flux. This also provides a possibility for the complete data prediction of subsequent membrane components and membrane fouling requirements.
(3) In the future research work, data are an important basis and resource for large data prediction research. Therefore, it is of strategic significance to establish a large database of membrane fouling standards for membrane components, to explore deep migration learning methods for predicting technological innovation, and to reveal the evolution mechanism of membrane fouling.

## Figures and Tables

**Table 1:** The SPSS analysis results.

Principal Component	Eigenvalues	Contribution Rate	Cumulative Contribution Rate
$Y_{1}$	2.331	53.752	53.752
$Y_{2}$	1.252	21.121	74.873
$Y_{3}$	1.007	14.435	89.308
$Y_{4}$	0.774	3.704	93.012

**Table 2:** Test function.

Test Function	Range of Values	Dimension	Optimum Solution
$F_{1}(x)=\sum_{i=1}^{n}x_{i}^{2}$	[−100,100]	30	0
$F_{2}(x)=\sum_{i=1}^{n}\left|x_{i}\right|+\prod_{i=1}^{n}\left|x_{i}\right|$	[−10,10]	30	0
$F_{3}(x)=\sum_{i=1}^{n}{\left(\sum_{j-1}^{i}x_{j}\right)}^{2}$	[−100,100]	30	0
$F_{4}(x)=\max_{i}\left\{\left|x_{i}\right|,1\leq i\leq n\right\}\;$	[−100,100]	30	0
$F_{5}(x)=\sum_{i=1}^{n}ix_{i}^{4}+\mathrm{random}[0,1)$	[−1.28,1.28]	30	0
$F_{6}(x)=\sum_{i=1}^{n}\left[x_{i}^{2}-10\cos\left(2\pi x_{i}\right)+10\right]\;$	[−5.12,5.12]	30	0
$F_{7}(x)=-20\exp\left(-0.2\sqrt{\frac{1}{n}\sum_{i=1}^{n}x_{i}^{2}}\right)-\exp\left(\frac{1}{n}\sum_{i=1}^{n}\cos\left(2\pi x_{i}\right)\right)+20+e$	[−32,32]	30	0
$F_{8}(x)=\frac{1}{4000}\sum_{i=1}^{n}x_{i}^{2}-\prod_{i=1}^{n}\cos\left(\frac{x_{i}}{\sqrt{i}}\right)+1$	[−600,600]	30	0

**Table 3:** Test function results.

Benchmark Function	SSA		Tent-SSA		PSO		WOA	
	Mean	Std. Deviation	Mean	Std. Deviation	Mean	Std. Deviation	Mean	Std. Deviation
$F_{1}$	1.73 × 10^−06^	2.66 × 10^−05^	**2.28 × 10** **^−164^**	**0**	5.26 × 10^−04^	7.32 × 10^−04^	2.01 × 10^−27^	1.11 × 10^−26^
$F_{2}$	6.32 × 10^−25^	1.35 × 10^−24^	**2.04 × 10** **^−184^**	**0**	2.35 × 10^−03^	1.61 × 10^−03^	2.44 × 10^−19^	4.56 × 10^−19^
$F_{3}$	−1.39 × 10^−06^	2.73 × 10^−05^	**8.02 × 10** **^−179^**	**0**	6.52 × 10^+^^02^	4.83 × 10^+^^02^	6.55 × 10^+^^03^	1.76 × 10^+^^03^
$F_{4}$	−3.74 × 10^−06^	1.02 × 10^−05^	**0**	**0**	6.13 × 10^+00^	2.25 × 10^+00^	6.36 × 10^+^^01^	2.53 × 10^+^^01^
$F_{5}$	2.43 × 10^−02^	1.01 × 10^−01^	**2.04 × 10** **^−02^**	**2.36 × 10** **^−03^**	4.84 × 10^−02^	1.74 × 10^−02^	6.13 × 10^−03^	8.20 × 10^−03^
$F_{6}$	9.95 × 10^−02^	1.13 × 10^+00^	**−1.04 × 10** **^−27^**	**1.39 × 10** **^−28^**	6.22 × 10^+01^	1.75 × 10^−01^	6.04 × 10^+00^	2.00 × 10^+01^
$F_{7}$	−1.11 × 10^−07^	3.31 × 10^−06^	**2.04 × 10** **^−20^**	**2.64 × 10** **^−20^**	8.04 × 10^−01^	8.01 × 10^−01^	8.25 × 10^−06^	2.52 × 10^−06^
$F_{8}$	9.37 × 10^−03^	8.33 × 10^−03^	**4.85 × 10** **^−11^**	**1.58 × 10** **^−10^**	8.26 × 10^−01^	8.83 × 10^−01^	9.28 × 10^−02^	6.24 × 10^−02^

**Table 4:** Prediction error comparison.

Model	EVA		
	MAPE/%	RMSE	MAE
BP	0.0216	0.3917	0.5148
GA-BP	0.0051	0.0344	0.1249
PSO-BP	0.0053	0.0484	0.1333
SSA-ELM	0.0046	0.0317	0.1157
SSA-BP	0.0024	0.0204	0.0574
Tent-PSO-BP	0.0025	0.0211	0.0606
Tent-SSA-BP	0.0007	0.0009	0.0226

**Table 5:** Prediction accuracy of different methods under different noise conditions.

Diagnostic Method	SNR/dB		
	4	8	12
BP	46.52%	63.82%	45.23%
GA-BP	82.51%	90.76%	79.96%
PSO-BP	84.67%	89.26%	80.42%
SSA-ELM	82.43%	88.17%	78.17%
SSA-BP	90.26%	92.28%	86.73%
Tent-PSO-BP	88.36%	90.23%	85.18%
Tent-SSA-BP	93.74%	94.16%	91.22%

## Data Availability

The data sources used in our experiments have been added in reference [19].
